# The cross‐sectional association of White Matter Hyperintensities, CAIDE Dementia Risk Score, and cognition in Mexican American elders from the HABS‐HD Study

**DOI:** 10.1002/alz.089499

**Published:** 2025-01-09

**Authors:** Raul Vintimilla

**Affiliations:** ^1^ University of North Texas Health Science Center, Fort Worth, TX USA

## Abstract

**Background:**

Associations between CAIDE scores and white matter hyperintensities (WMH) have been reported, and research have shown that both can impact cognition. The cognitive correlates of high CAIDE scores and WMH load in minorities like Mexican Americans (MA) remain unclear.

**Method:**

Cognitively normal MA from the Health and Aging Brain Study (HABS‐HD) (n = 112), were classified as high or low risk for dementia according to a CAIDE cutoff score of 9. WMH volume was measured from FLAIR using the Statistical Parametric Mapping Lesion Segmentation Tool. After comparing global cognition, memory, executive function (EF), verbal fluency, and processing speed (PS) scores between groups, generalized linear models were used to explore the associations of WMH, CAIDE score and cognition. To better understand the association of WMH and CAIDE score with cognitive performance, we conducted a mediation analysis using PROCESS Macro (Hayes 2013). Age, sex, and education were entered as covariates in the models.

**Result:**

Participants in the high‐risk group were older, less educated, and had lower scores in all cognitive measures. CAIDE score was not associated with any cognitive measures. WMH were associated with memory (B = ‐.14, p <0.001), EF (B = ‐.13, p = .002), and PS (B = .89, p = .02), and the three measures were also associated with the interaction high risk x WMH. In mediation analysis, the regression of WMH on the mediator, CAIDE, was significant (b = ‐.57, p <.001). The analysis showed that the mediator (CAIDE), controlling for WMH, was significant for executive function (b = ‐.09, p <0.001), and processing speed (b = ‐.03, p = .002). The analyses also revealed that, controlling for the mediator (CAIDE), WMH was still a significant predictor of executive function (b = ‐.18, p <0.001), and processing speed (b = ‐.15, p <0.001), but the effects were smaller.

**Conclusion:**

These results suggest that MA with higher WMH perform worse in measures of executive function and processing speed, and that association is partially mediated by higher CAIDE scores. Further studies are needed to investigate if cognitive performance in normal individuals with WMH and high CAIDE scores.

